# Depression symptoms, anxiety, and stress among undergraduate entrance admission seeking students in Bangladesh: a cross-sectional study

**DOI:** 10.3389/fpubh.2023.1136557

**Published:** 2023-04-26

**Authors:** Md. Reza-A Rabby, Md. Saiful Islam, Maisha Tahsin Orthy, Ahmad Tousif Jami, M. Tasdik Hasan

**Affiliations:** ^1^Department of Educational & Counseling Psychology, University of Dhaka, Dhaka, Bangladesh; ^2^BRAC Institute of Educational Development, BRAC University, Dhaka, Bangladesh; ^3^Department of Public Health and Informatics, Jahangirnagar University, Dhaka, Bangladesh; ^4^Centre for Advanced Research Excellence in Public Health, Dhaka, Bangladesh; ^5^Department of Psychology, University of Dhaka, Dhaka, Bangladesh; ^6^School of Liberal Arts and Social Sciences, School of Business and Entrepreneurship, Independent University, Dhaka, Bangladesh; ^7^Action Lab, Department of Human Centred Computing, Faculty of Information Technology, Monash University, Melbourne, VIC, Australia; ^8^Department of Public Health, State University of Bangladesh, Dhaka, Bangladesh

**Keywords:** depression, anxiety, stress, university admission, young students, Bangladesh

## Abstract

**Background:**

Intense academic pressure and unhealthy competition have turned the university entrance exam scenario in Bangladesh into a Pandora's Box, which might cause mental health difficulties among young students. However, there is a severe scarcity of studies concerning such issues of university entrance examination-seeking students in Bangladesh.

**Methods:**

This study aimed to assess the prevalence and associated factors of depression symptoms, anxiety, and stress among undergraduate entrance admission-seeking students in Bangladesh. A cross-sectional study design was followed using an online tool including socio-demographic questions, and the 21-item Bangla Depression, Anxiety and Stress Scale (BDASS-21). The survey form was completed by 452 Bangladeshi students who passed the higher secondary certificate (HSC) examination in 2020 and were planning to get admission to the undergraduate level during the data collection.

**Results:**

The prevalence of mild to extremely severe levels of depression symptoms, anxiety, and stress was 57.7%, 61.4%, and 44.6%, respectively. Females were more likely to have depression, anxiety, and stress symptoms than males. The students from science backgrounds were at higher risk of developing depression and stress symptoms when compared with students from business studies backgrounds. Besides, students with a previous history of mental illness, a preference for getting admitted into the public university, and less monthly family income (<25,000 BDT) were more likely to develop depression, anxiety, and stress symptoms. In addition, students with a previous history of neurological disorders were more likely to develop anxiety symptoms than those without.

**Conclusion:**

This study revealed a high level of depression, anxiety, and stress symptoms among undergraduate entrance admission-seeking students, which calls for in-depth exploratory investigations. Adequate low-intensity interventions should be designed to support this young population.

## Background

Mental health concerns are emerging as a significant public health challenge in low- and middle-income countries such as Bangladesh (1). According to a systematic review, the prevalence of mental disorders in Bangladesh ranged from 6.5 to 31.0% for adults (18–65 years), and 13.4 to 22.9% for children (5–17 years) (2). And to make it worse, mental health services in Bangladesh are severely compromised due to a lack of skilled mental health professionals. The Bangladesh government only spends 0.44% of its overall health budget on mental health. Only 0.11% of people have free access to the necessary psychiatric help (3).

Students' mental health is a legitimate concern all over the world. Globally students have a high prevalence of depression, anxiety, and stress symptoms (4–7). These can have a detrimental effect on the individual. The examination can be a source of anxiety for students who are preparing for the university entrance examination. Students in Turkey were found with a high prevalence of university entrance test anxiety which is around 48.1% (8). Exams can become a major cause of stress, especially when they function as a criterion for future prospects and professional paths (9, 10). Exam-related anxiety and stress have a negative impact on student's academic performance, physical health and development, and quality of life (4). Bangladeshi youths were found with a negative impact on their mental health in terms of stress, anxiety, and depression due to examination (11). In Bangladesh, university entrance examinations are particularly important and also have a significant impact on career choices. Students preparing for these exams are trained in private educational institutions. They set aside an expenditure for it based on socioeconomic capabilities.

The university entrance exam in Bangladesh is a competitive examination where the students who have completed their secondary and higher secondary studies need to seat for competitive tests to get selected for their desired educational institution. Each undergraduate university conducts the examinations separately and the students need to prepare themselves rigorously for these tests. However, universities can only admit a small number of students who are meritoriously qualified in the entrance tests due to very limited seats (about 51-thousand seats in public universities) (12). These create overwhelming challenges for them considering the time they receive to get prepared. Subsequently, students who are preparing for such a challenging entrance examination go through a major change in their lifestyle.

In the year 2020, Bangladeshi government was unable to organize the Higher Secondary Certificate (HSC) and equivalent exams due to the COVID-19 pandemic. Hence, all 13, 67,377 students who took the HSC or an equivalent exam were successful in obtaining their certificates without the formal examination process. About 11.83 % of these students earned a GPA-5 of (Grade Point Average−5, highest GPA), nearly tripling the percentage from the previous year (13). The undergraduate entrance admission-seeking candidates had to wait longer than usual because of the pandemic stresses and altered administration decisions. This potentially resulted in heightened mental health difficulties among the students. Additionally, this specific cohort of students faced social neglect by the neighbors and even family members as they received the auto pass status without attending the test which might be a factor contributing to their poor mental health (13).

Some studies provided evidence regarding the poor mental health condition of Bangladeshi university students (14, 15). Additionally, Bangladeshi medical students show a very high frequency of depression symptoms, with the COVID-19 pandemic underway, the concerning prevalence and associated variables of depression point to the necessity for follow-up psychological interventions targeted at medical students (16). Safa et al. (17) found that 27.3% of medical students have mild anxiety, 26.8% have moderate anxiety, and 11.8% have severe anxiety.

To the best of the authors' knowledge, there was no such published report at the time of conceptualization of the present study that described the mental health difficulties of university entrance examination-seeking students in Bangladesh. We suspect the effect of the university entrance exam on the mental health of examinees may impact them lifelong, for this phase of their life is one of the most challenging. Thus, the present study aimed to investigate the prevalence and associated factors of depression symptoms, anxiety, and stress among undergraduate entrance admission-seeking students in Bangladesh during a period of global uncertainties and overwhelming challenges.

## Methods and materials

### Study design and participants

A web-based cross-sectional survey was conducted among undergraduate entrance admission seeking students in Bangladesh from April 25 to July 28, 2021. Data were collected from both male and female Bangladeshi students (18 years and above) who passed the Higher Secondary Certificate (HSC) examination in 2020 and planning to get admission to university settings during the time of the data collection. There was no restriction on which part of the country they belong to or which course they are planning to get admission into. Data was collected through an online survey using the convenience sampling technique.

The sample size is calculated from the prevalence estimate using the following formula (18):


$$\begin{array}{l} n=\frac{z^{2}p\left(1-p\right)}{d^{2}} \end{array}$$

where, *n* = number of samples, *z* = 1.96 for 95% confidence level (CI), *p* = “best guess” for prevalence, and *d* = precision of the prevalence estimate. We assumed that the prevalence of mental health difficulties among undergraduate admission test examinees might be 50%. The calculated sample size was 384 participants. Assuming a 15% non-response rate, we calculated the sample size as 442. However, a total of 466 participants took part in the study. About 14 data were discarded due to incomplete submission. In order to acquire more precise results, 452 data were included in the final analyses.

### Measurement tool

The online assessment tools that were used in this survey had three sections. The first section included details of the survey followed by an electronic consent form; the second part consists of socio-demographic variables such as sex, age, education group (Science/Arts/ Business studies), grade point average (GPA) of Secondary School Certificate (SSC) and Higher Secondary Certificate (HSC; later categorized as 4–5 and 3–3.99), residence (rural/urban), desired university type (public/ private), family income range, and two additional questions. The first additional question was whether or not the participant had any previous mental disorder-related history. The second additional question was whether or not the participant had any previously diagnosed neurological disorder-related history. The third section includes the validated Bangla version of the Depression, Anxiety, and Stress Scale (DASS-21), which had Cronbach's Alpha scores for Depression, Anxiety, and Stress subscales of 0.987, 0.957, 0 964 for Depression, Anxiety, and Stress subscales respectively (19). The original DASS-21 is a 21-item self-report scale composed of three self-report scales (depression, anxiety, and stress) (20). The DASS-21 indicated satisfactory internal consistency with a Cronbach's alpha score of 0.92 (21). The results of each sub-total scale will fall into one of five categories: minimal, mild, moderate, severe, or extremely severe. It's a four-point Likert scale that considers the activity and feelings of the most recent week. The Bangla DASS-21 is a significant tool for assessing Bangladeshis' psychological wellbeing (22). In the present study, the overall Cronbach's alpha of the DASS-21 scale was 0.96; and the Cronbach's alpha for the depression, anxiety, and stress subscales were 0.92, 0.88, and 0.91, respectively.

### Procedure

After getting approval from the *Ethical Review Committee of the Public Health Foundation, Bangladesh (PHF, BD*; ref. no. 03/2021), the data collection was initiated via online methods using Google Forms owing to considering the pandemic situation. The research team distributed the survey link through social media to participants from all administrative divisions of Bangladesh. The survey link was also shared on social media across numerous university entrance-seeking student forums (e.g., Facebook groups). The survey form of the present study was also uploaded to the Facebook groups of the HSC 2020 batch as they are university admission seekers. It was an open and voluntary survey where no incentives were provided. The technical functionality of the electronic questionnaire was tested before implementing the questionnaire. After giving the electronically written instructions on what to do and ensuring confidentiality, the participants were asked to go through the three parts of the survey form. The procedure followed the Checklist for Reporting Results of Internet E-surveys (CHERRIES) guideline (23). All procedures used in this study follow the ethical standards of the relevant national and institutional human experimentation ethical guidelines and the Helsinki Declaration of 1975, as revised in 2008.

### Statistical analysis

Some descriptive analyses (e.g., frequencies, percentages, means, and standard deviations) were performed to determine the characteristics of the participants. The relationship between DASS subscale scores and other studied variables was also predicted using bivariate and multivariable linear regression. The variables that were significant in the bivariate analysis were modeled separately for depression, anxiety, and stress in the multivariable regression analysis. A *p*-value of 0.05 was deemed significant. Two statistical packages of software (i.e., IBM SPSS Statistics version 25.0 and STATA version 14.0) were used for all types of statistical analyses.

## Results

### Characteristics of the participants

A total of 452 participants (60.18% males; mean age = 19.31 ± 0.98 years) were included in the final analysis. Of them, most were unmarried (89.38%), and almost half had a science background (48.67%). Sizeable participants expressed their preference to get admitted into public universities (75.44%). Most had Grade Point Average (GPA) between 4 and 5 in their higher secondary certificate exam (90.04%), while nearly equal participants (90.49%) had GPA between 4 and 5 in their secondary school certificate exam. More than one-third (42.92%) had monthly family income between 25,000 and 50,000 BDT (Bangladeshi Taka; ≈ 300–600 USD), and nearly three-fourth lived in urban areas (70.13%). A small group of participants reported previous history of mental (7.3%) and neurological (8.41%) disorders (Table 1).

**Table 1:** General characteristics of the participants (*N* = 452).

**Variables**	***n* (%)**
**Sex**	
Male	272 (60.18)
Female	180 (39.82)
**Age**	
18–20 years	400 (88.5)
21–24 years	52 (11.5)
**Study major**	
Science	220 (48.67)
Arts	157 (34.73)
Business studies	75 (16.59)
**HSC GPA**	
3–3.99	45 (9.96)
4–5	407 (90.04)
**SSC GPA**	
3–3.99	43 (9.51)
4–5	409 (90.49)
**Preferred university for undergrad**	
Public	341 (75.44)
Private	111 (24.56)
**Marital status**	
Married	48 (10.62)
Unmarried	404 (89.38)
**Residence**	
Urban	317 (70.13)
Rural	135 (29.87)
**Monthly family income**	
<25,000	104 (23.01)
25,000–50,000	194 (42.92)
50,001–100,000	131 (28.98)
>100,000	23 (5.09)
**Mental disorder history**	
Yes	33 (7.3)
No	419 (92.7)
**Neurological disorder history (e.g., epilepsy**,	
**migraine, Alzheimer's)**	
Yes	38 (8.41)
No	414 (91.59)

### Depression symptoms

**Figure 1:**
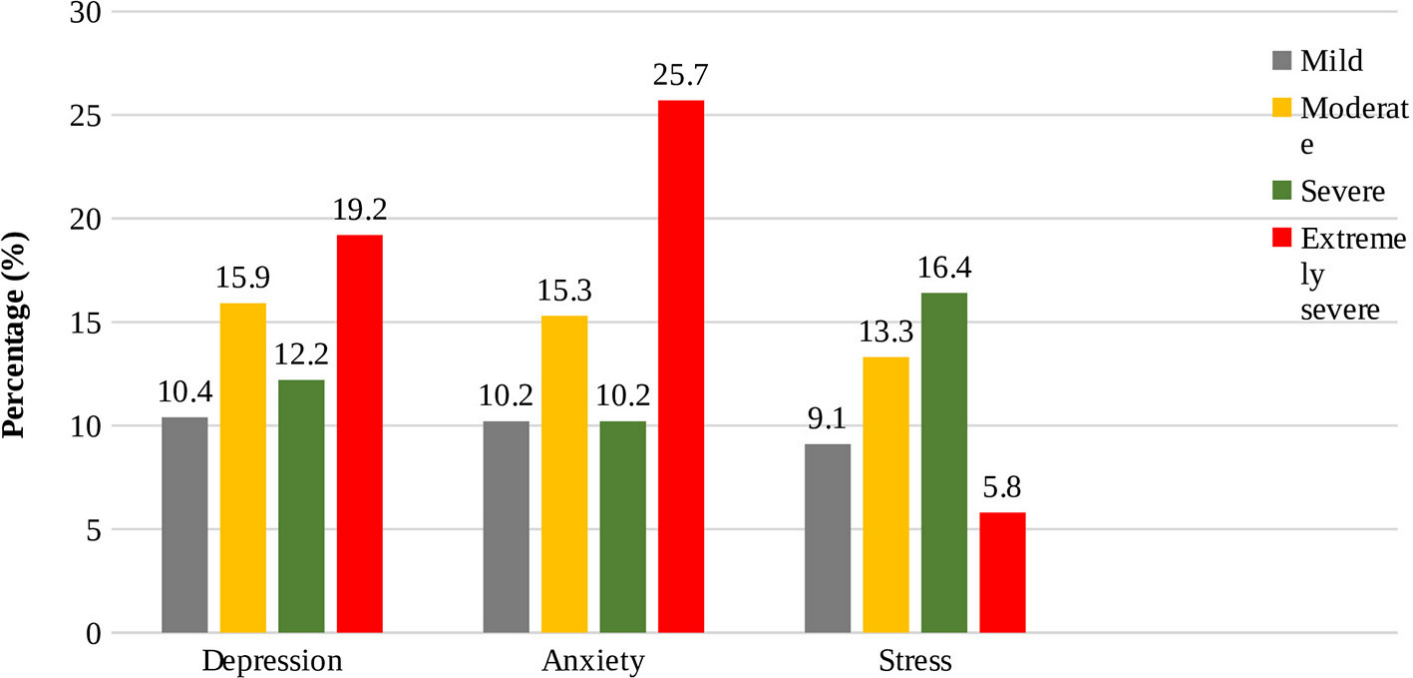
The level of respondents (*n* = 452) experiencing varying levels of depression, anxiety, and stress.

The prevalence estimates of mild, moderate, severe, and extremely severe depression were 10.4%, 15.9%, 12.2%, and 19.2%, respectively (Figure 1). As per as multiple linear regression analysis (Table 2), the positively predicting factors of depression score included: (i) being female (β = 0.18, *p* < 0.001), (ii) having “science” major (β = 0.16, *p* = 0.011) in reference to “business studies”, (iii) having “public” as preferred university for undergrad (β = 0.18, *p* < 0.001), and (iv) having mental disorder history (β = 0.19, *p* < 0.001). Whereas, the negatively predicting factor of depression score was having monthly family income “25,000–50,000 BDT” (β = −0.13, *p* = 0.026) in reference to “ <25,000 BDT.”

**Table 2:** Bivariate and multivariable regression analyses by depression score.

**Variables**	**Mean (SD)**	**Unadjusted estimate**					**Adjusted estimate**				
		***B***	**SE**	***t***	β	***p*** **-value**	***B***	**SE**	***t***	β	***p*** **-value**
**Sex**											
Female	17.76 (12.71)	4.29	1.12	3.84	0.18	<0.001	4.26	1.05	4.05	0.18	<0.001
Male	13.46 (10.86)	Ref.					Ref.				
**Age**											
21–24 years	17.73 (12.51)	2.89	1.74	1.66	0.08	0.097					
18–20 years	14.84 (11.69)	Ref.									
**Study major**											
Science	17.85 (12.64)	5.06	1.54	3.28	0.21	0.001	3.82	1.49	2.56	0.16	0.011
Arts	12.55 (10.1)	−0.25	1.62	−0.16	−0.01	0.876	0.57	1.57	0.36	0.02	0.720
Business studies	12.8 (10.93)	Ref.					Ref.				
**HSC GPA**											
4–5	15.53 (11.9)	3.57	1.85	1.93	0.09	0.054					
3–3.99	11.96 (10.47)	Ref.									
**SSC GPA**											
4–5	15.5 (11.97)	3.46	1.89	1.83	0.09	0.068					
3–3.99	12.05 (9.62)	Ref.									
**Preferred university for undergrad**											
Public	16.55 (12.16)	5.59	1.26	4.42	0.02	<0.001	4.93	1.27	3.89	0.18	<0.001
Private	10.95 (9.53)	Ref.					Ref.				
**Marital status**											
Unmarried	15.52 (11.91)	3.27	1.80	1.82	0.09	0.070					
Married	12.25 (10.56)	Ref.									
**Residence**											
Urban	15.46 (11.93)	0.98	1.21	0.80	0.04	0.422					
Rural	14.49 (11.52)	Ref.									
**Monthly family income (BDT)**											
25,000–50,000	13.16 (11.56)	−4.49	1.42	−3.16	−0.19	0.002	−3.06	1.37	−2.23	−0.13	0.026
50,001–100,000	15.91 (11.61)	−1.75	1.54	−1.14	−0.07	0.257	0.14	1.49	0.10	0.01	0.924
>100,000	16.7 (14.84)	−0.96	2.70	−0.36	−0.02	0.722	−0.22	2.59	−0.09	<-0.01	0.931
<25,000	17.65 (11.3)	Ref.					Ref.				
**Mental disorder history**											
Yes	23.82 (11.8)	9.33	2.09	4.46	0.21	<0.001	8.74	2.19	3.99	0.19	<0.001
No	14.49 (11.55)	Ref.					Ref.				
**Neurological disease history (e.g., epilepsy, migraine, Alzheimer's)**											
Yes	19.68 (12.03)	4.93	1.99	2.48	0.12	0.014	1.90	2.03	0.93	0.04	0.352
No	14.76 (11.71)	Ref.					Ref.				

### Anxiety

The prevalence estimates of mild, moderate, severe, and extremely severe anxiety were 10.2%, 15.3%, 10.2%, and 25.7%, respectively (Figure 1). As per as multiple linear regression analysis (Table 3), the positively predicting factors of anxiety score included: (i) being female (β = 0.22, *p* < 0.001), (ii) having “public” as the preferred university for undergrad (β = 0.13, *p* < 0.001), (iii) having mental disorder history (β = 0.22, *p* < 0.001), and (iv) having neurological disorder history (β = 0.10, *p* = 0.039). Whereas, the negatively predicting factor of anxiety score was having a monthly family income of “25,000–50,000 BDT” (β = −0.15, *p* = 0.009) in reference to “ <25,000 BDT.”

**Table 3:** Bivariate and multivariable regression analyses by anxiety score.

**Variables**	**Mean (SD)**	**Unadjusted estimate**					**Adjusted estimate**				
		***B***	**SE**	***t***	β	***p*** **-value**	***B***	**SE**	***t***	β	***p*** **-value**
**Sex**											
Female	15.22 (10.71)	4.44	0.94	4.74	0.22	<0.001	4.39	0.89	4.96	0.22	<0.001
Male	10.79 (9.03)	Ref.					Ref.				
**Age**											
21–24 years	13.65 (10.6)	1.24	1.47	0.85	0.04	0.397					
18–20 years	12.41 (9.88)	Ref.									
**Study major**											
Science	13.93 (10.6)	2.97	1.32	2.24	0.15	0.025	1.84	1.26	1.47	0.09	0.143
Arts	11.39 (9.35)	0.43	1.39	0.31	0.02	0.758	0.88	1.32	0.66	0.04	0.507
Business studies	10.96 (8.76)	Ref.					Ref.				
**HSC GPA**											
4–5	12.71 (9.9)	1.60	1.57	1.02	0.05	0.307					
3–3.99	11.11 (10.46)	Ref.									
**SSC GPA**											
4–5	12.67 (9.95)	1.23	1.60	0.77	0.04	0.442					
3–3.99	11.44 (10.08)	Ref.									
**Preferred university for undergrad**											
Public	13.35 (9.91)	3.24	1.08	3.00	0.14	0.003	3.08	1.07	2.88	0.13	0.004
Private	10.11 (9.75)	Ref.					Ref.				
**Marital status**											
Unmarried	12.63 (9.95)	0.71	1.52	0.47	0.02	0.640					
Married	11.92 (10.11)	Ref.									
**Residence**											
Urban	13 (9.99)	1.49	1.02	1.45	0.07	0.147					
Rural	11.51 (9.84)	Ref.									
**Monthly family income**											
25,000–50,000	11.07 (10)	−3.62	1.20	−3.01	−0.18	0.003	−3.01	1.16	−2.61	−0.15	0.009
50,001–100,000	13.18 (9.52)	−1.52	1.30	−1.17	−0.07	0.243	−0.23	1.25	−0.18	−0.01	0.853
>100,000	11.83 (10.85)	−2.87	2.28	−1.26	−0.06	0.209	−2.57	2.18	−1.18	−0.06	0.239
<25,000	14.69 (9.9)	Ref.					Ref.				
**Mental disorder history**											
Yes	21.39 (9.89)	9.54	1.75	5.46	0.25	<0.001	8.49	1.85	4.60	0.22	<0.001
No	11.86 (9.64)	Ref.					Ref.				
**Neurological disorder history (e.g., epilepsy, migraine, Alzheimer's)**											
Yes	18.42 (11.57)	6.41	1.66	3.85	0.18	<0.001	3.55	1.71	2.07	0.10	0.039
No	12.01 (9.64)	Ref.					Ref.				

### Stress symptoms

The prevalence estimates of mild, moderate, severe, and extremely severe stress were 9.1%, 13.3%, 16.4%, and 5.8%, respectively (Figure 1). As per as multiple linear regression analysis (Table 4), the positively predicting factors of stress score included: (i) being female (β = 0.22, *p* < 0.001), (ii) having “science” major (β = 0.14, *p* = 0.021) in reference to “business studies,” (iii) having “public” as preferred university for undergrad (β = 0.19, *p* < 0.001), and (iv) having mental disorder history (β = 0.21, *p* < 0.001). Whereas, the negatively predicting factor of stress score was having monthly family income “25,000–50,000 BDT” (β = −0.19, *p* = 0.001) in reference to “ <25,000 BDT.”

**Table 4:** Bivariate and multivariable regression analyses by stress score.

**Variables**	**Mean (SD)**	**Unadjusted estimate**					**Adjusted estimate**				
		***B***	**SE**	***t***	β	***p*** **-value**	***B***	**SE**	***t***	β	***p*** **-value**
**Sex**											
Female	17.78 (11.63)	4.93	1.03	4.79	0.22	<0.001	4.89	0.95	5.13	0.22	<0.001
Male	12.85 (10.03)	Ref.					Ref.				
**Age**											
21–24 years	16.19 (11.62)	1.56	1.62	0.96	0.05	0.335					
18–20 years	14.63 (10.87)	Ref.									
**Study major**											
Science	17.14 (11.19)	4.76	1.44	3.32	0.22	0.001	3.14	1.35	2.32	0.14	0.021
Arts	12.73 (10.44)	0.35	1.51	0.23	0.02	0.815	0.73	1.43	0.51	0.03	0.611
Business studies	12.37 (9.99)	Ref.					Ref.				
**HSC GPA**											
4–5	15.09 (10.88)	2.78	1.72	1.62	0.08	0.106					
3–3.99	12.31 (11.43)	Ref.									
**SSC GPA**											
4–5	15.07 (10.9)	2.65	1.75	1.51	0.07	0.132					
3–3.99	12.42 (11.28)	Ref.									
**Preferred university for undergrad**											
Public	16.19 (10.87)	5.59	1.17	4.78	0.22	<0.001	4.83	1.15	4.20	0.19	<0.001
Private	10.59 (10.16)	Ref.					Ref.				
**Marital status**											
Unmarried	15.15 (10.99)	3.15	1.67	1.89	0.09	0.060					
Married	12 (10.3)	Ref.									
**Residence**											
Urban	15.05 (10.79)	0.80	1.13	0.71	0.03	0.477					
Rural	14.25 (11.35)	Ref.									
**Monthly family income**											
25,000–50,000	12.77 (11.04)	−5.53	1.31	−4.23	−0.25	<0.001	−4.25	1.24	−3.42	−0.19	0.001
50,001–100,000	15.28 (10.5)	−3.03	1.42	−2.14	−0.13	0.033	−1.17	1.35	−0.87	−0.05	0.386
>100,000	13.57 (11.88)	−4.74	2.48	−1.91	−0.10	0.057	−4.05	2.34	−1.73	−0.08	0.085
<25,000	18.31 (10.36)	Ref.					Ref.				
**Mental disorder history**											
Yes	23.52 (9.61)	9.39	1.93	4.86	0.22	<0.001	8.89	1.99	4.47	0.21	<0.001
No	14.13 (10.77)	Ref.					Ref.				
**Neurological disorder history (e.g., epilepsy, migraine, Alzheimer's)**											
Yes	19.95 (11.45)	5.60	1.84	3.05	0.14	0.002	2.51	1.84	1.36	0.06	0.174
No	14.34 (10.8)	Ref.					Ref.				

## Discussion

The present study aimed to investigate the prevalence of depression symptoms, anxiety, and stress among undergraduate admission-seeking students of Bangladesh and to identify the associated factors related to depression symptoms, stress, and anxiety. According to the responses of undergraduate entrance-seeking participants, 57.7% of students reported mild to extremely severe depression symptoms, 61.4% of students reported mild to extremely severe anxiety, and 44.6% of students reported mild to extremely severe stress (Figure 1), indicating a higher prevalence rate of stress, anxiety, and depression symptoms in comparison with a countrywide study of student mental health in Bangladesh (14). The prevalence of depression, anxiety, and stress among students were lower than a larger spectrum of students in the present study in contrast to the prior Bangladeshi studies (19, 24, 25). This discrepancy in the prevalence rate of our study may be due to the difference in the geographical area or education level of the participants from where they were sampled. Also, the pandemic factor might be played a role in this discrepancy.

We further divided the discussion with relevant subheadings for a clear understanding as follows.

### Depression symptoms

A study that estimated the prevalence of depression in Bangladeshi adolescent students discovered that 61.3% of students experienced depression symptoms, which is higher than what we observed in this study. Age range or the use of different scales to assess levels of depression may result in discrepancies in the study findings (26).

### Anxiety

The prevalence rate of anxiety was 61.4% among our study participants, indicating a higher prevalence rate of anxiety levels before the undergraduate entrance exam compared to a similar kind of study with the students of Turkey who were going to take the university entrance examination (8).

### Stress

The prevalence of stress in the current study was lower than in one Egyptian study when compared to the prevalence levels of stress using the DASS globally [62.4% (27)] but higher than in another Malaysian study [23.7% (28)].

### Severity of symptoms

Our findings reveal that undergraduate admission-seeking students in Bangladesh suffer from serious mental health issues, which is consistent with the findings by Roy et al. (29) that indicated uncertainties regarding academic progress for young individuals can be problematic. This may be higher due to factors such as a lack of social or familial support or both, fears about the future, a toxic psychosocial environment, the extent of the exam curriculum, and the limited amount of preparation time available (4, 30).

### Gender

In the present study, females were more like to have higher depression symptoms, anxiety, and stress scores, which is consistent with the findings of several international studies (28, 31). Possible causes include the enormous rise in teen marriage and gender-based violence against women since the beginning of the pandemic in 2020 (25). This result also loosely supports the study which is conducted with Turkish students, where the female population showed more severe stress symptoms compared to male participants (4) while contradicting few other studies which suggest that male students are more likely than female students to experience a high level of depression, anxiety and stress (32, 33).

### Relationship status

The present study showed no significant difference among participants in their depression symptoms, anxiety, and stress levels regarding their marital status, which is somewhat in line with the findings by Marthoenis et al. (34), which found that marital status has no relevance to the occurrence of anxiety symptoms. This result shows disagreement with findings from several studies that claim unmarried people are more likely to experience depression, anxiety, and stress (27, 35), as well as with the findings of Hossain et al. (25) that claimed that being in a committed relationship was found to be a predictor of experiencing higher levels of anxiety and stress.

### Academic background

The present findings indicated a higher level of depression and stress scores among the participants studying in the science group compared to students who were from the business studies background, which is slightly consistent with the findings of a prior study conducted with ZagaZig University students that participants in the faculty of pharmacy reported more depression symptoms than students in other faculties (36). Students in the sciences may experience more obstacles and competition, which might lead to mental health issues. This result is in parallel with the findings of several studies conducted among physicians and medical students in Bangladesh, which also indicated the high prevalence of anxiety, stress, and depression symptoms among them (37, 38).

### Aim for public vs. private universities

The present study found that students aiming for public universities at their undergrad level have more depression, anxiety, and stress symptoms than those who seek private universities. None of the previous studies investigated this association. This may result from the excessive study load they go through during their admission-seeking days before the undergrad entrance test for the public university. In addition, parental or family expectations and pressures for getting admitted into public universities might worsen the condition though this influence needs further exploration.

### Income status

In the present study, participants with lower monthly family income (>25,000 BDT) showed more depression symptoms, anxiety, and stress. A survey of Turkish university students found similar results in terms of depression that students from low-income families had higher depression scores than those from wealthier families (4). Another longitudinal study by Lorant et al. (39) supports our observations that students in the lowest socioeconomic group are more likely to experience depression than those in the highest socioeconomic group, even though a separate study indicated no connection between the socioeconomic status of the family and students' depression, anxiety, and stress levels (28). The high prevalence of depression, anxiety, and stress symptoms among students from low-income families may be related to the pressure that is often brought on by financial hardship (40). In a systematic review, Lund et al. (41) also found that poverty is associated with greater rates of mental illness in low and middle-income nations (LMIC) like Bangladesh.

### Previously diagnosed mental disorders

Students diagnosed with previous mental disorders were more likely to have more depression, anxiety, and stress symptoms than those who did not have such experience. In contrast, students with a history of previous neurological disorders were more likely to have anxiety symptoms than those who did not have such experience. Both of these findings are consistent with Bass et al. (1) that mental and neurological disorders profoundly impact the mental wellbeing of individuals.

### Parent-children relationship

The high rates of depression, anxiety, and stress among Bangladeshi undergraduate entrance exam students may be attributable to several factors which include trouble interacting, family and societal pressure, high parental expectations, parental conflicts, insufficient financial assistance and difficulties, concerns about the future, a toxic psychological environment, a large admission test curriculum, and massive test timeframes (33). Furthermore, it is considered that when parents get too concerned in children's lives, it might hinder the growth of their autonomy, resulting in a diminished sense of personal capability or mastery and an increased awareness that the world is “uncontrollable” (42). The assertion is that all of these things add up to a strenuous environment for students. Caster et al. (43) observed that anxious students evaluated their parents as more socially isolated, preoccupied with others' judgments, and unhappy with their poor performance. Therefore, it may be possible to reduce the prevalence of mental health problems related to entrance examinations among undergraduate entrance admission-seeking students in Bangladesh by strengthening the parent-children relationship. Students struggling with exam-related anxiety may engage in suicidal behavior if they fail the exam (44). One suicide due to failing the medical college entrance exam in 2021 drew nationwide public attention, which indicated a critical need to look into the current mental health difficulties among Bangladeshi undergraduate entrance admission-seeking students (45).

### COVID-19 pandemic

High rates of depression, anxiety, and stress may be partially explained by the timing of our study, which coincided with the COVID-19 pandemic. They may feel pressed by their delayed undergraduate entry admittance test, which they have not taken or have had to reschedule owing to the pandemic. Agitation, hostility, and regressive behavior are just some of the issues that could emerge or worsen as a result of that. There will soon be widespread emotional discomfort and an elevated risk of mental health issues because of the imposed measures that restrict their freedoms during the pandemic in response to that unknown diseases with unpredictable prognoses (46).

Fountoulakis et al. (47) discovered that a sizable percentage of persons experienced a decline in their mental health, family relationships, and general quality of life during the COVID-19 epidemic. Fountoulakis et al. (47) created the *COMET-G model*, which uncovered multiple vulnerabilities and an interplay that can lead to clinical depression and suicidal ideation through distress. As our study was conducted during the pandemic, considering the model, this particular dynamics might have played a critical role to develop symptoms that could have been investigated though we missed this opportunity to capture this critical data adequately.

### Limitations

There were some limitations to this study. First, it was self-reported, which has specific limitations than a clinically diagnosed study. Second, there was a chance of recall bias as it was impossible to validate the information due to the study's cross-sectional design. The participants only included internet users, which can cause a bias in the perceived result. As the study was cross-sectional in nature, the causal relationships between depression, anxiety, stress symptoms, and associated factors cannot be established. Finally, the present study used the convenience sampling technique, which may not reflect the actual condition of the whole population.

Further studies are needed using more representative samples to get generalizable findings for the entire population. Acknowledging these limitations, it can be argued that this study reported some novel findings from a tender population group and calls for in depths explorations of the factors associated with these mental health difficulties faced by the young students.

## Conclusion

The current study revealed high depression symptoms, anxiety, and stress among Bangladeshi undergraduate admission-seeking students. Being a female student, having a lower monthly family income, preferring to attend a public university, and having a previous history of mental disorders were factors associated with experiencing adverse mental health symptoms among this young population. Furthermore, majoring in science was related to the development of depression and stress symptoms, whereas a history of neurological disorders was associated with the development of anxiety symptoms. These critical and novel findings could aid in the design of specific mental health interventions for this subset of the population. Furthermore, these findings may be used to develop appropriate student-friendly, empathetic community or family-focused strategies and action plans to support the mental health of this vulnerable group engaging their families. This also calls for revisiting the admission examination process, creating an additional burden on the students.

## Data availability statement

The raw data supporting the conclusions of this article will be made available by the authors, without undue reservation.

## Ethics statement

The studies involving human participants were reviewed and approved by Ethical Review Committee of the Public Health Foundation, Bangladesh. The patients/participants provided their written informed consent to participate in this study.

## Author contributions

The study was conceptualized by MR. The theoretical background and methodology portions were developed by MR, MO, and MH. The data was collected by MR, MO, and AJ. The data was evaluated by MR and MI. The discussion parts were written by MR, MO, and MI. MH contributed as the senior author. All authors provided feedback on the manuscript draft. All authors contributed to the article and approved the submitted version.
